# Internet Addiction, Mental Health Help-Seeking, and Mental Health Problems Among Chinese Adolescents: Repeated Cross-Sectional Study

**DOI:** 10.2196/90014

**Published:** 2026-08-12

**Authors:** Afei Qin, Meiqi Wang, Lianlong Yu, Suyun Li, Zhaolu Liu, Shoujuan Zheng, Ziming Shao, Cuixia Lv, Long Sun

**Affiliations:** 1 Department of Social Medicine and Health Management, School of Public Health, Cheeloo College of Medicine, Shandong University, 44 Wenhuaxi Road, Jinan, Shandong, 250012, China, 86 0531-88382678, 86 13256660556; 2 NHC Key Lab of Health Economics and Policy Research (Shandong University), Jinan, Shandong, China; 3 Center for Health Management and Policy Research, Shandong University (Shandong Provincial Key New Think Tank), Jinan, Shandong, China; 4 School (Institute) of Mental Health and Psychological Sciences, Cheeloo College of Medicine, Shandong University, Jinan, Shandong, China; 5 Shandong Center for Disease Control and Prevention, Jinan, Shandong, China; 6 School of Public Health, Cheeloo College of Medicine, Shandong University, Jinan, Shandong, China; 7 School of Public Health, Shandong First Medical University & Shandong Academy of Medical Sciences, Jinan, Shandong, China; 8 School of Public Health, Shandong Second Medical University, Weifang, Shandong, China

**Keywords:** internet addiction, mental health help-seeking, depressive symptoms, anxiety symptoms, adolescents

## Abstract

**Background:**

Internet addiction (IA) has been consistently associated with adverse mental health outcomes, but less is known about whether adolescents with IA seek mental health support, and whether associations between help-seeking and mental health problems differ across pathways.

**Objective:**

This study aimed to describe mental health help-seeking patterns across internet use and IA status, and examine the independent and interactive associations of IA and help-seeking with mental health problems.

**Methods:**

Data were drawn from 2 repeated cross-sectional surveys conducted in Shandong Province in 2023 and 2024. A total of 171,970 junior middle school students were included. IA was assessed using a 9-item scale adapted from *DSM-5* (*Diagnostic and Statistical Manual of Mental Disorders* [Fifth Edition]) Internet Gaming Disorder criteria to assess internet use–related symptoms. Students using the internet for at least 4 hours per day and endorsing at least 4 symptoms were classified as having IA. Help-seeking was categorized as no help-seeking, informal-only help-seeking, and any formal help-seeking. Mental health problems were defined as clinically significant depressive or anxiety symptoms. Pairwise logistic regression compared help-seeking patterns, and Poisson regression with school-clustered CR2 robust SEs estimated adjusted prevalence ratios (PRs).

**Results:**

Overall, 1.4% (2406/171,970) met the definition of IA; 47.9% (82,402/171,970), 42.1% (72,461/171,970), and 9.9% (17,107/171,970) reported no help-seeking, informal-only help-seeking, and any formal help-seeking, respectively. Among adolescents with IA, 59.9% (1440/2406) reported no help-seeking, 33.8% (814/2406) reported informal-only help-seeking, and only 6.3% (152/2406) reported any formal help-seeking. Compared with nonaddictive internet users, adolescents with IA had lower odds of informal-only versus no help-seeking (OR [odds ratio] 0.690, 95% CI 0.631‐0.754), any formal versus no help-seeking (OR 0.540, 95% CI 0.455‐0.640), and any formal versus informal-only help-seeking (OR 0.786, 95% CI 0.659‐0.937). Compared with nonaddictive internet users, adolescents with IA had a higher prevalence of mental health problems (PR 3.662, 95% CI 3.428‐3.912). Informal-only (PR 0.581, 95% CI 0.550‐0.614) and any formal help-seeking (PR 0.461, 95% CI 0.420‐0.505) were associated with lower prevalence overall. Interaction terms for IA with informal-only help-seeking (PR 1.469, 95% CI 1.336‐1.616) and any formal help-seeking (PR 2.033, 95% CI 1.696‐2.437) indicated weaker inverse associations among adolescents with IA. Within the IA group, informal-only help-seeking was associated with lower prevalence (PR 0.848, 95% CI 0.784‐0.917), whereas any formal help-seeking was not clearly associated with lower prevalence (PR 0.945, 95% CI 0.814‐1.097). Sensitivity analyses supported these patterns.

**Conclusions:**

Adolescents with IA had a substantially higher prevalence of clinically significant depressive and anxiety symptoms and were less likely to report mental health help-seeking, especially formal help-seeking. These findings highlight a high-burden, low help-seeking subgroup and suggest strengthening school-based mental health services, improving linkage between informal and formal support, reducing stigma, and developing low-threshold digital mental health pathways.

## Introduction

With the rapid spread of the internet and digital technologies, online activities have become deeply embedded in adolescents’ daily lives and social interactions. A growing body of evidence suggests that the psychological effects of internet use among adolescents are not a simple dichotomy of “harmful” versus “harmless,” but may vary by the intensity, purpose, and context of use [1-3]. However, excessive and uncontrolled internet use accompanied by impaired control or functional impairment may indicate internet addiction (IA). IA is commonly defined as a form of behavioral addiction, characterized by strong urges to use the internet and impaired control over use, resulting in significant academic, social, or functional impairment [4,5]. An increasing number of studies have consistently shown that IA is closely associated with a range of adverse mental health outcomes, including depression, loneliness, sleep problems, and suicidal ideation or attempts [6-14]. With an increasingly earlier age of onset and a continuously rising prevalence, IA has gradually become an important public health issue associated with a substantial mental health burden among adolescents and sustained challenges to both educational and public health systems.

Given that IA has been consistently associated with a higher prevalence of mental health problems among adolescents, understanding how adolescents cope with psychological distress is particularly important. Mental health help-seeking is generally defined as the process by which individuals actively seek support from informal or professional sources when experiencing psychological distress [15]. Informed by the theory of planned behavior, help-seeking intentions and behaviors may be shaped by adolescents’ attitudes toward seeking help, perceived social norms, and perceived behavioral control [16]. Adolescents’ willingness to seek help is influenced by multiple factors, including individual-level mental health literacy and coping styles, social-level stigma, trust, and emotional support, as well as structural-level accessibility and availability of services [17]. In addition, adolescents with more severe psychological symptoms may paradoxically show lower help-seeking intention, as depression and related barriers such as helplessness, shame, stigma, and emotional exhaustion may suppress help-seeking [17,18]. However, among adolescents with IA, who are at particularly high risk for mental health problems, it remains unclear whether psychological distress can be translated into actual help-seeking behavior, and whether patterns of choice differ between formal and informal help-seeking pathways. This uncertainty represents a key gap in understanding the persistence of mental health risks and identifying opportunities for timely support.

In the Chinese context, this research gap is particularly evident. Although adolescent mental health has received increasing attention at the policy level in recent years, formal mental health services continue to face multiple structural and cultural barriers. First, the overall number of mental health professionals is insufficient and unevenly distributed, with particularly severe shortages in rural areas and primary health care settings [19-21]. Second, although school-based mental health services have been increasingly emphasized, existing prevention and intervention practices still face regional disparities, incomplete pathways from screening to classification and intervention, and limited integration across service components [22]. Third, stigma and negative attitudes toward professional psychological help remain important barriers. Evidence from Chinese middle school students with depressive symptoms suggests low professional help-seeking and higher self-stigma among those with depressive symptoms [23]. In addition, fragmentation in policy frameworks and financing mechanisms undermines the affordability and continuity of mental health services [24]. In this context, informal help-seeking, such as seeking support from family members, teachers, or peers, occupies a central position in China’s adolescent mental health support system and, for many adolescents, becomes the primary or even the only source of help. For example, a school-based survey conducted in Hunan Province reported that informal sources accounted for 99.3% of adolescents’ help-seeking behaviors [25]. However, within this context of heavy reliance on informal help-seeking, whether different help-seeking pathways are differentially associated with mental health problems among adolescents with IA remains insufficiently examined.

Junior middle school students represent a key early-adolescent group in which internet use habits, emotional problems, and help-seeking behaviors are rapidly developing, while school-based support systems are becoming increasingly important. Based on the aforementioned research gaps, this study has the following aims. First, we describe patterns of mental health help-seeking across different levels of internet use and IA, and clarify differences among adolescents with IA in terms of whether they seek help and their choice between formal and informal help-seeking pathways. Second, we examine the independent associations of IA and mental health help-seeking behaviors with mental health problems. Furthermore, we investigate the interaction between IA and different types of mental health help-seeking behaviors to assess whether the association between IA and mental health problems differs by help-seeking pathway. Overall, we aim to clarify the help-seeking behavioral patterns of adolescents with IA and their implications for mental health, thereby providing empirical evidence for enhancing school-based, multilevel mental health support systems that integrate formal and informal resources. By identifying adolescents with high IA-related mental health burden and low help-seeking, the findings may also inform the design and targeting of digital mental health strategies, including school-based digital screening, online help-seeking pathways, and blended digital-offline support systems that connect adolescents to informal and formal mental health resources.

## Methods

### Data Sources and Study Population

Data for this study were obtained from the Surveillance and Intervention Project for Common Diseases and Health Influencing Factors among Students in Shandong Province. This project is part of a nationally coordinated school health surveillance system led by provincial Centers for Disease Control and Prevention, aiming to systematically monitor common diseases and health-related factors among children and adolescents and to provide evidence for policymaking and service planning. It is a repeated, large-scale cross-sectional surveillance survey. Details of this surveillance system have been described elsewhere [26]. Since 2023, the questionnaire has included newly added modules on mental health help-seeking and anxiety, providing key variables for this study. Although the surveillance system collected data from students across multiple school stages, this study was restricted a priori to junior middle school students to focus on early adolescence and to reduce heterogeneity in developmental stage, school context, academic pressure, internet use patterns, and help-seeking resources. The surveillance in Shandong Province covers urban and suburban areas across all 16 prefecture-level cities, targeting students from fourth grade in primary school to third year in university.

### Sampling and Survey Procedures

**Figure 1.**
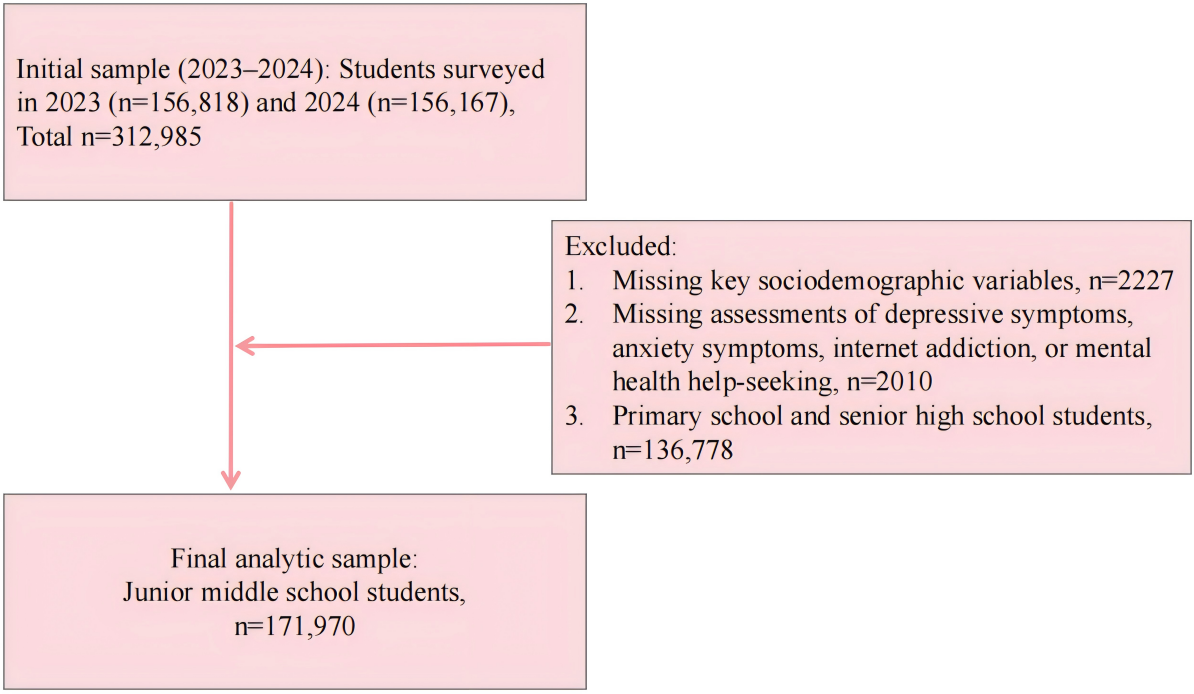
Participant inclusion and exclusion flowchart for the 2023 and 2024 surveillance surveys.

A stratified cluster random sampling strategy was used, with the province, prefecture-level city, and county (district) serving as the 3 sampling strata. In each urban district, 8 schools were randomly selected (including 2 primary schools, 2 junior middle schools, 2 general high schools, 1 secondary vocational school, and 1 comprehensive university). In each county, 5 schools were randomly selected (including 2 primary schools, 2 junior middle schools, and 1 general high school), and 2 additional kindergartens were sampled for myopia surveillance. Monitoring schools were generally kept consistent across survey years to allow for temporal comparisons. Within each selected junior middle school, classes served as the sampling units. At least 2 classes were randomly selected from each grade, and all students in selected classes were invited to participate. Each grade included at least 80 surveyed students, and each school’s total sample size was not fewer than 240 students. When the sample size of a selected school was insufficient, a school of the same type within the same district or county was sampled as a replacement. Data were collected through anonymous, self-administered electronic or paper questionnaires, supervised onsite by trained investigators during classroom sessions. The Shandong Provincial Center for Disease Control and Prevention was responsible for data review, entry, and quality control to ensure representativeness, reliability, and consistency. A total of 156,818 and 156,167 students were surveyed in 2023 and 2024, respectively. After applying the screening procedure presented in Figure 1, the final analytic sample comprised 171,970 junior middle school students.

### Internet Usage and IA

IA was assessed using a 9-item scale adapted from the *DSM-5* (*Diagnostic and Statistical Manual of Mental Disorders* [Fifth Edition]) symptom criteria for Internet Gaming Disorder [27]. Because Internet Gaming Disorder specifically pertains to gaming, the items were modified to refer to general internet use across devices and online activities, including mobile phones, tablets, computers, and other internet-enabled devices. Therefore, this measure should be interpreted as a screening measure for internet use–related addiction-like symptoms rather than a clinical diagnosis of Internet Gaming Disorder. Students self-reported whether they had experienced relevant symptoms during the past week. Students were first asked whether they had accessed the internet, followed by a question regarding their average daily online time during the past 7 days. The symptom assessment comprised 9 items, listed in Methods S1 in Multimedia Appendix 1. These items measured withdrawal symptoms (items 1 and 2), tolerance (item 3), escape behaviors (items 4, 6, and 9), impaired control (items 5 and 8), and denial (item 7). Each item had 2 response options (“yes” or “no”), with “yes” indicating the presence of the symptom. The 9 symptom items were summed to create an IA symptom score ranging from 0 to 9, with higher scores indicating more endorsed symptoms. Following a previous Chinese adolescent surveillance study using this operational definition, students were classified as having IA if they used the internet for at least 4 hours per day and endorsed at least 4 of the 9 symptoms [28]. The scale demonstrated good internal consistency in this study (Cronbach α=0.808) and has been applied among Chinese adolescents [28].

### Mental Health Help-Seeking

Mental health help-seeking among adolescents was measured using 2 questions. First, students were asked, “Have you ever sought help from others when you were troubled by psychological problems?” with response options of “yes” and “no.” This item assessed whether students had ever sought help and did not specify a recent time frame. For those who answered “yes,” we further assessed the specific sources from which they sought help using a multiple-choice item. The options covered both informal social support and professional help resources, including (1) parents, (2) teachers, (3) classmates or friends, (4) online sources, (5) school psychological counseling office, (6) psychological support hotline, and (7) hospital services. Among adolescents who reported seeking help, those who selected one or more of options 1‐4 only were classified as having informal mental health help-seeking. Those who selected one or more of options 5‐7 were classified as having any formal mental health help-seeking, regardless of whether they also selected informal sources. Accordingly, adolescents in this study were categorized into 3 groups: no help-seeking, informal-only help-seeking, and any formal help-seeking. To evaluate the influence of overlap between informal and formal sources, sensitivity analyses reclassified help-seeking into 4 mutually exclusive categories: no help-seeking, informal-only help-seeking, formal-only help-seeking, and both informal and formal help-seeking.

### Mental Health Problems

Mental health problems were defined based on whether participants displayed clinically significant depressive or anxiety symptoms. Depressive symptoms were assessed using the 20-item Center for Epidemiologic Studies Depression Scale (CESD-20), which has been widely used in epidemiological studies and has shown acceptable psychometric performance among Chinese adolescents [29,30]. Total scores range from 0 to 60, with higher scores indicating more severe symptoms. In accordance with established cut-offs, a score of ≥16 was used to define clinically significant depressive symptoms [29]. Anxiety symptoms were measured using the 7-item Generalized Anxiety Disorder Scale (GAD-7), which has been validated in Chinese adolescents [31]. Total scores range from 0 to 21, and a score of ≥10 was used to define clinically significant anxiety symptoms [32].

### Covariates

Covariates included survey year (2023 or 2024), sex (male or female), age (years), grade, monitoring site (urban or suburban county), ethnicity (Han or other minority), boarding status (yes or no), history of chronic disease, myopia status (proxied by wearing corrective glasses), and BMI. Students reported whether they had been diagnosed with any of the following chronic conditions in the previous year: hepatitis, nephritis, heart disease, hypertension, anemia, diabetes, allergic diseases, or physical disability. The presence of at least one of these conditions was classified as a history of chronic disease. BMI was categorized as thinness, normal weight, overweight, and obesity. Height was measured using a mechanical stadiometer (accurate to 0.1 cm), and weight was measured using an electronic scale (accurate to 0.1 kg). Overweight and obesity were defined according to the Screening for Overweight and Obesity among School-age Children and Adolescents (WS/T 586‐2018) [33]. Thinness was defined according to the Screening for Malnutrition among School-age Children and Adolescents (WS/T 456‐2014), with “mild” and “moderate-to-severe” thinness combined [34].

### Data Analysis

Statistical analyses were conducted using R software (version 4.3.0; R Foundation for Statistical Computing). The analytic dataset was constructed using a complete-case approach, retaining participants with complete data on school identifier, survey year, IA status, mental health help-seeking, mental health outcomes, and all covariates included in the adjusted models. Continuous variables were summarized as means and SDs, and categorical variables as frequencies and percentages. Help-seeking patterns were described across IA status using cross-tabulations. To compare help-seeking categories across IA status, pairwise logistic regression models with school-clustered CR2 robust SEs were fitted for 3 contrasts: informal-only help-seeking versus no help-seeking, any formal help-seeking versus no help-seeking, and any formal help-seeking versus informal-only help-seeking. Exponentiated coefficients were reported as odds ratios (ORs) for these help-seeking category contrasts, with 95% CIs.

Because mental health problems were relatively common, Poisson regression models with a log link were used as the primary analytic approach to estimate adjusted prevalence ratios (PRs) and 95% CIs for binary outcomes [35]. The primary model included IA status and mental health help-seeking simultaneously, with nonaddictive internet use and no help-seeking specified as the reference categories. Models were adjusted for survey year, sex, age, grade, monitoring site, ethnicity, boarding status, chronic disease history, myopia status, and BMI category. School-clustered CR2 robust SEs were used to account for within-school correlation. Mental health problems were analyzed as the main outcome, and depressive symptoms and anxiety symptoms were analyzed separately in supplementary models using the same framework. Logistic regression models with school-clustered CR2 robust SEs were fitted as secondary analyses to provide ORs, but PRs were used as the main effect estimates.

To examine whether the association between IA status and mental health problems differed by help-seeking category, multiplicative interaction models were fitted by adding an IA status × help-seeking term to the Poisson regression models. Interaction terms were interpreted on the multiplicative PR scale. To facilitate interpretation from the help-seeking perspective, additional Poisson models were fitted within each IA status group, with no help-seeking as the reference category. We also created a 9-category joint exposure variable combining the 3 IA status categories with the 3 help-seeking categories. In this joint-category analysis, nonaddictive internet use plus no help-seeking was used as the reference group. Adjusted PRs, 95% CIs, cell counts, and observed prevalence of mental health problems were used to present the joint pattern of IA status, help-seeking, and mental health problems, including in the forest plot. Benjamini-Hochberg adjustment was applied to prespecified pairwise comparisons, and 2-sided adjusted *P* values <.05 were considered statistically significant.

Several supplementary analyses were conducted to examine whether the main findings were consistent across alternative outcome specifications and internet use measures. Depressive symptoms and anxiety symptoms were first analyzed separately as binary outcomes using the same Poisson regression framework, including main-effect models, interaction models, and stratified help-seeking models within each IA status. We also tabulated the observed prevalence of mental health outcomes and mean symptom scores across the 9 joint categories of IA status and help-seeking. In addition, daily internet use duration was analyzed as an alternative internet use measure using 4 categories: no internet use, less than 2 hours per day, 2 to 4 hours per day, and 4 hours or more per day. Continuous CESD-20 and GAD-7 scores were further analyzed using linear regression models with school-clustered CR2 robust SEs. Details of these supplementary analyses are provided in Methods S2 in Multimedia Appendix 1.

Several sensitivity analyses were performed to assess the robustness of the findings. To address uncertainty in the IA definition, we repeated the analyses using alternative binary definitions based on different combinations of internet use duration and symptom thresholds, including symptom-only definitions and definitions excluding potentially affect-laden items. Continuous IA symptom scores were also analyzed, including the full 9-item score and scores excluding item 9 or items 1, 2, and 9. To examine the potential influence of the repeated cross-sectional design, single-year analyses were conducted separately for the 2023 and 2024 survey waves. Finally, to evaluate the overlap between informal and formal help-seeking sources, help-seeking was reclassified into 4 mutually exclusive categories, and an additional analysis excluded adolescents with formal-only help-seeking. All sensitivity analyses used the same covariate adjustment strategy and school-clustered CR2 robust SEs as the main models. Full specifications and results are presented in Methods S3 in Multimedia Appendix 1.

Data analysis and reporting followed the STROBE (Strengthening the Reporting of Observational Studies in Epidemiology) guidelines (Checklist 1).

### Ethical Considerations

This study complied with the Declaration of Helsinki and relevant Chinese regulations. Ethical approval was obtained from the Medical Ethics Committee of the Shandong Center for Disease Control and Prevention (approval number SDJK(K)2025-057-01). All participants or their legal guardians provided written informed consent; for those unable to sign, oral consent was documented in the presence of an independent witness.

## Results

### Overview

A total of 171,970 junior middle school students were included in the analysis, of whom 84,509 (49.1%) were surveyed in 2023 and 87,461 (50.9%) in 2024. Boys accounted for 53.1% (91,340/171,970) of the sample, and the mean age was 13.49 (SD 1.03) years. Overall, 41.1% (70,587/171,970) of students lived in urban areas and 58.9% (101,383/171,970) in suburban counties; 98.7% (169,742/171,970) were of Han ethnicity. Approximately 17.6% (30,209/171,970) of students boarded at school, 1.3% (2178/171,970) reported at least one chronic disease, and 45.7% (78,563/171,970) wore glasses. Regarding internet use, 47,196/171,970 (27.4%) students reported no internet use, 87,690/171,970 (51%) used the internet for less than 2 hours per day, 24,891/171,970 (14.5%) for 2‐4 hours per day, and 12,193/171,970 (7.1%) for 4 hours or more per day. The mean daily internet use duration was 1.67 (SD 2.50) hours, and the mean IA symptom score was 0.51 (SD 1.33). In total, 122,368/171,970 (71.2%) students were classified as nonaddictive internet users, 47,196/171,970 (27.4%) as noninternet users, and 2406/171,970 (1.4%) as having IA. For mental health help-seeking, 82,402/171,970 (47.9%) students reported no help-seeking, 72,461/171,970 (42.1%) reported informal-only help-seeking, and 17,107/171,970 (9.9%) reported any formal help-seeking. The overall prevalence of mental health problems (depression or anxiety) was 14.9%; the prevalence of depressive symptoms and anxiety symptoms was 14.3% and 5.1%, respectively. The mean CESD-20 and GAD-7 scores were 8.96 (SD 8.30) and 2.36 (SD 3.72), respectively (Table 1).

**Table 1.** Characteristics of participants and mental health outcomes (N=171,970)^a^.

Characteristics	Value
Year, n (%)	
2023	84,509 (49.14)
2024	87,461 (50.86)
Sex, n (%)	
Male	91,340 (53.11)
Female	80,630 (46.89)
Age (years), mean (SD)	13.49 (1.03)
Monitoring site, n (%)	
Urban	70,587 (41.05)
Suburban county	101,383 (58.95)
Ethnicity, n (%)	
Han	169,742 (98.70)
Others	2228 (1.30)
Grade, n (%)	
Grade 7	53,347 (31.02)
Grade 8	52,480 (30.52)
Grade 9	51,215 (29.78)
Fourth-year junior middle school^b^	14,928 (8.68)
Boarding at school, n (%)	
No	141,761 (82.43)
Yes	30,209 (17.57)
Number of chronic disease conditions, n (%)	
0	169,792 (98.73)
≥1	2178 (1.27)
Wearing glasses, n (%)	
No	93,407 (54.32)
Yes	78,563 (45.68)
BMI, n (%)	
Normal weight	100,856 (58.65)
Thinness	3192 (1.86)
Overweight	31,847 (18.52)
Obesity	36,075 (20.98)
Daily internet use duration (h/d), n (%)	
No internet use	47,196 (27.44)
<2	87,690 (50.99)
2‐4	24,891 (14.47)
≥4	12,193 (7.09)
Daily internet use duration (h/d), mean (SD)	1.67 (2.50)
Internet addiction status, n (%)	
No internet use	47,196 (27.44)
Nonaddictive internet use	122,368 (71.16)
Internet addiction	2406 (1.40)
Internet addiction symptom score, mean (SD)	0.51 (1.33)
Mental health help-seeking, n (%)	
No help-seeking	82,402 (47.92)
Informal-only help-seeking	72,461 (42.14)
Any formal help-seeking	17,107 (9.95)
Depressive symptoms, n (%)	
No	147,305 (85.66)
Yes	24,665 (14.34)
Anxiety symptoms, n (%)	
No	163,129 (94.86)
Yes	8841 (5.14)
CESD-20^c^ score, mean (SD)	8.96 (8.30)
GAD-7^d^ score, mean (SD)	2.36 (3.72)
Mental health problems, n (%)	
No	146,318 (85.08)
Yes	25,652 (14.92)

a Values are presented as n (%) for categorical variables and mean (SD) for continuous variables. Mental health problems were defined as the presence of clinically significant depressive symptoms (CESD-20 ≥16) or anxiety symptoms (GAD-7 ≥10). BMI categories follow WS/T 586-2018 for overweight/obesity and WS/T 456-2014 for thinness.

b In some areas of Shandong Province, compulsory education follows a 5+4 structure, with 5 years of primary school and 4 years of junior middle school, rather than the more common 6+3 structure. This category refers to students in the fourth and final year of junior middle school before entering senior high school.

c CESD-20: 20-item Center for Epidemiologic Studies Depression Scale.

d GAD-7: 7-item Generalized Anxiety Disorder Scale.

### IA Status and Mental Health Help-Seeking Patterns

#### Overview

Help-seeking patterns differed across IA status (Table 2). Among nonaddictive internet users, 47.4% (58,040/122,368) reported no help-seeking, 42.8% (52,325/122,368) reported informal-only help-seeking, and 9.8% (12,003/122,368) reported any formal help-seeking. Among noninternet users, the corresponding proportions were 48.6% (22,922/47,196), 40.9% (19,322/47,196), and 10.5% (4952/47,196). In contrast, adolescents with IA had a higher proportion of no help-seeking and lower proportions of both informal-only and any formal help-seeking: 59.9% (1440/2406) reported no help-seeking, 33.8% (814/2406) reported informal-only help-seeking, and only 6.3% (152/2406) reported any formal help-seeking.

**Table 2.** Internet addiction status and mental health help-seeking patterns^a^.

Variables	No help-seeking, n (%)	Informal-only help-seeking, n (%)	Any formal help-seeking, n (%)	Informal-only vs no help-seeking, OR^b^ (95% CI)	Any formal vs no help-seeking, OR (95% CI)	Any formal vs informal-only, OR (95% CI)
Reference: nonaddictive internet use						
Nonaddictive internet use	58,040 (47.43)	52,325 (42.76)	12,003 (9.81)	1 (reference category)	1 (reference category)	1 (reference category)
Internet addiction	1440 (59.85)	814 (33.83)	152 (6.32)	0.690 (0.631‐0.754)^c^	0.540 (0.455‐0.640)^c^	0.786 (0.659‐0.937)^d^
Reference: no internet use						
No internet use	22,922 (48.57)	19,322 (40.94)	4952 (10.49)	1 (reference category)	1 (reference category)	1 (reference category)
Nonaddictive internet use	58,040 (47.43)	52,325 (42.76)	12,003 (9.81)	1.122 (1.096‐1.149)^c^	1.031 (0.992‐1.072)	0.928 (0.893‐0.965)^e^
Internet addiction	1440 (59.85)	814 (33.83)	152 (6.32)	0.774 (0.707‐0.847)^c^	0.557 (0.468‐0.661)^c^	0.729 (0.610‐0.871)^e^

a Any formal help-seeking refers to seeking help from a school counseling service, psychological hotline, or hospital, regardless of whether informal help was also sought. Odds ratios were obtained from pairwise logistic regression models comparing the specified help-seeking categories. Models used pairwise logistic regression with school-clustered CR2 robust SEs and adjusted for survey year, sex, age, grade, monitoring site, ethnicity, boarding status, chronic disease history, myopia status, and BMI category. Benjamini-Hochberg–adjusted *P* values were used for pairwise comparisons of internet addiction status.

b OR: odds ratio.

c *P<*.001.

d *P*<.05.

e *P*<.01.

In adjusted pairwise logistic comparisons using nonaddictive internet use as the reference group, adolescents with IA had lower odds of reporting informal-only help-seeking rather than no help-seeking (OR 0.690, 95% CI 0.631‐0.754), and lower odds of reporting any formal help-seeking rather than no help-seeking (OR 0.540, 95% CI 0.455‐0.640). When any formal help-seeking was compared directly with informal-only help-seeking, adolescents with IA also had lower odds of reporting any formal help-seeking (OR 0.786, 95% CI 0.659‐0.937). Similar patterns were observed when no internet use was used as the reference category. The distribution of individual help-seeking sources further showed that parents were the most frequently reported source of help, followed by classmates or friends and teachers, whereas school counseling offices, psychological hotlines, and hospital services were less commonly reported (Table S1 in Multimedia Appendix 1).

#### Associations of IA Status and Help-Seeking With Mental Health Problems

In the main-effect Poisson regression model, IA status was strongly associated with mental health problems (Table 3). Compared with nonaddictive internet users, noninternet users had a lower prevalence of mental health problems (9.70% vs 15.96%; adjusted PR 0.625, 95% CI 0.586‐0.667), whereas adolescents with IA had a substantially higher prevalence (64.42%; adjusted PR 3.662, 95% CI 3.428‐3.912). Mental health help-seeking was also associated with mental health problems. Compared with adolescents reporting no help-seeking, those reporting informal-only help-seeking had a lower prevalence of mental health problems (11.30% vs 19.39%; adjusted PR 0.581, 95% CI 0.550‐0.614), as did those reporting any formal help-seeking (8.68%; adjusted PR 0.461, 95% CI 0.420‐0.505).

**Table 3.** Associations of internet addiction status and help-seeking with mental health problems^a^.

Variable and category	Mental health problems, n/N (%)	Adjusted PR^b^ (95% CI)
Internet addiction status		
Nonaddictive internet use	19,524/122,368 (15.96)	1 (reference category)
No internet use	4578/47,196 (9.70)	0.625 (0.586‐0.667)^c^
Internet addiction	1550/2406 (64.42)	3.662 (3.428‐3.912)^c^
Mental health help-seeking		
No help-seeking	15,980/82,402 (19.39)	1 (reference category)
Informal-only help-seeking	8187/72,461 (11.30)	0.581 (0.550‐0.614)^c^
Any formal help-seeking	1485/17,107 (8.68)	0.461 (0.420‐0.505)^c^

a Poisson regression with log link and school-clustered CR2 robust SEs was used. Models adjusted for survey year, sex, age, grade, monitoring site, ethnicity, boarding status, chronic disease history, myopia status, and BMI category. Mental health problems were defined as CESD-20 (20-item Center for Epidemiologic Studies Depression Scale) ≥16 or GAD-7 (7-item Generalized Anxiety Disorder Scale) ≥10.

b PR: prevalence ratio.

c *P*<.001.

Supplementary models examining depressive symptoms and anxiety symptoms separately showed similar patterns. IA was associated with a higher prevalence of depressive symptoms (PR 3.714, 95% CI 3.475‐3.969) and anxiety symptoms (PR 5.546, 95% CI 4.963‐6.197). Informal-only and any formal help-seeking were associated with lower prevalence of depressive symptoms and anxiety symptoms in the overall sample, although the magnitude differed between depressive and anxiety outcomes. The corresponding interaction models for depressive and anxiety symptoms were generally consistent with the interaction pattern observed for the composite mental health problem outcome (Tables S2-S5 in Multimedia Appendix 1). Logistic regression models yielded similar directions of association but larger effect estimates, and were therefore retained as secondary analyses.

#### Interaction and Stratified Associations

In the interaction model, the association between IA status and mental health problems differed by help-seeking category (Table 4). Among adolescents with no help-seeking, IA was associated with a markedly higher prevalence of mental health problems compared with nonaddictive internet use (PR 3.186, 95% CI 2.958‐3.431). Informal-only help-seeking and any formal help-seeking were associated with lower prevalence among nonaddictive internet users, with PRs of 0.584 (95% CI 0.550‐0.619) and 0.462 (95% CI 0.419‐0.510), respectively. However, interaction terms for IA with informal-only help-seeking (PR 1.469, 95% CI 1.336‐1.616) and with any formal help-seeking (PR 2.033, 95% CI 1.696‐2.437) were both greater than 1. These results indicate that the inverse association between help-seeking and mental health problems was weaker among adolescents with IA than among nonaddictive internet users. Secondary logistic regression estimates are presented in Table S6 in Multimedia Appendix 1.

**Table 4.** Interaction between internet addiction status and mental health help-seeking on mental health problems^a^.

Variables	PR^b^ (95% CI)	*P* value
Internet addiction status		
Nonaddictive internet use	1 (reference category)	—^c^
No internet use	0.663 (0.607‐0.725)	<.001
Internet addiction	3.186 (2.958‐3.431)	<.001
Mental health help-seeking		
No help-seeking	1 (reference category)	—
Informal-only help-seeking	0.584 (0.550‐0.619)	<.001
Any formal help-seeking	0.462 (0.419‐0.510)	<.001
Internet addiction status × mental health help-seeking		
Nonaddictive internet use × no help-seeking	1 (reference category)	—
No internet use × informal only help-seeking	0.857 (0.756‐0.973)	.017
Internet addiction × informal only help-seeking	1.469 (1.336‐1.616)	<.001
No internet use × any formal help-seeking	0.793 (0.617‐1.018)	.071
Internet addiction × any formal help-seeking	2.033 (1.696‐2.437)	<.001

a Mental health problems were defined as clinically significant depressive symptoms or anxiety symptoms. Poisson regression models with school-clustered CR2 robust SEs were used. Models adjusted for survey year, sex, age, grade, monitoring site, ethnicity, boarding status, chronic disease history, myopia status, and BMI category.

b PR: prevalence ratio.

c Not applicable.

The stratified analyses further clarified this pattern (Table 5). Among nonaddictive internet users, both informal-only help-seeking (PR 0.583, 95% CI 0.550‐0.618) and any formal help-seeking (PR 0.462, 95% CI 0.420‐0.509) were associated with lower prevalence of mental health problems compared with no help-seeking. Similar associations were observed among noninternet users, with PRs of 0.501 (95% CI 0.440‐0.570) for informal-only help-seeking and 0.367 (95% CI 0.288‐0.467) for any formal help-seeking. Among adolescents with IA, informal-only help-seeking was associated with a modestly lower prevalence of mental health problems (PR 0.848, 95% CI 0.784‐0.917), whereas any formal help-seeking was not clearly associated with lower prevalence (PR 0.945, 95% CI 0.814‐1.097). The number of adolescents with IA who reported any formal help-seeking was small (n=152), so the corresponding stratified estimate should be interpreted cautiously.

**Table 5.** Stratified associations of help-seeking with mental health problems within each internet addiction status^a^.

Internet addiction status and help-seeking category	Mental health problems, n/N (%)	PR^b^ (95% CI)	*P* value
Nonaddictive internet use			
No help-seeking	11,965/58,040 (20.62)	1 (reference category)	—^c^
Informal-only help-seeking	6415/52,325 (12.26)	0.583 (0.550‐0.618)	<.001
Any formal help-seeking	1144/12,003 (9.53)	0.462 (0.420‐0.509)	<.001
No internet use			
No help-seeking	3041/22,922 (13.27)	1 (reference category)	—
Informal-only help-seeking	1295/19,322 (6.70)	0.501 (0.440‐0.570)	<.001
Any formal help-seeking	242/4952 (4.89)	0.367 (0.288‐0.467)	<.001
Internet addiction			
No help-seeking	974/1440 (67.64)	1 (reference category)	—
Informal-only help-seeking	477/814 (58.60)	0.848 (0.784‐0.917)	<.001
Any formal help-seeking	99/152 (65.13)	0.945 (0.814‐1.097)	.46

a Models used Poisson regression with school-clustered CR2 robust SEs and adjusted for survey year, sex, age, grade, monitoring site, ethnicity, boarding status, chronic disease history, myopia status, and BMI category. The reference group for internet addiction status was nonaddictive internet use, and the reference group for help-seeking was no help-seeking.

b PR: prevalence ratio.

c Not applicable.

**Figure 2.**
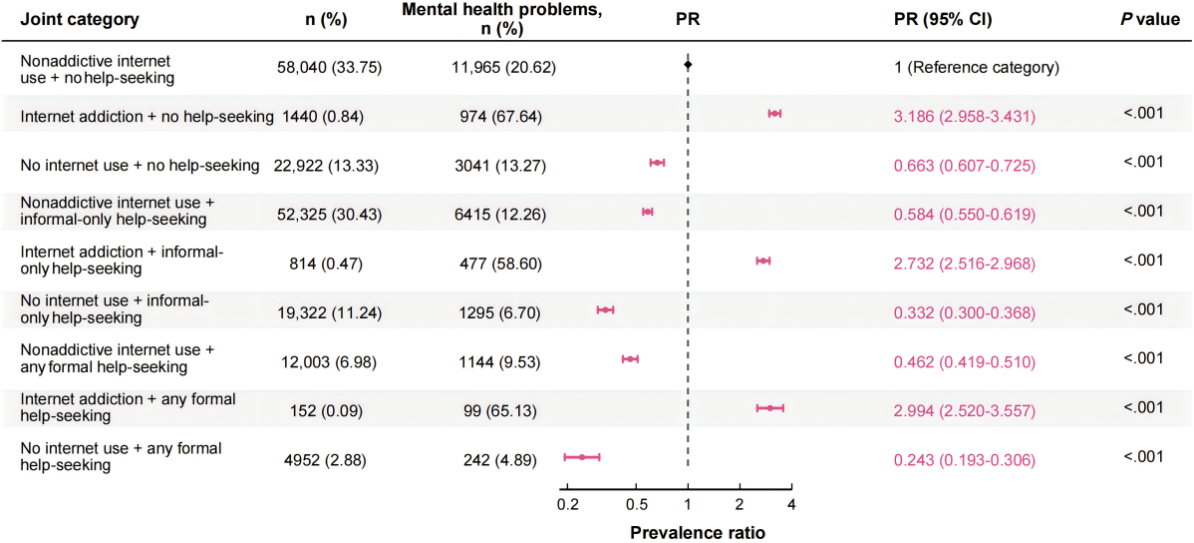
Joint categories of internet addiction status and mental health help-seeking in relation to mental health problems. Poisson regression models with school-clustered CR2 robust SEs were used. Models adjusted for survey year, sex, age, grade, monitoring site, ethnicity, boarding status, chronic disease history, myopia status, and BMI category. PR: prevalence ratio.

The 9-category joint analysis provided a more direct view of the combined pattern of IA status and help-seeking (Figure 2). Compared with nonaddictive internet users with no help-seeking, adolescents with IA consistently had a substantially higher prevalence of mental health problems regardless of help-seeking category. The adjusted PR was 3.186 (95% CI 2.958‐3.431) for IA with no help-seeking, 2.732 (95% CI 2.516‐2.968) for IA with informal-only help-seeking, and 2.994 (95% CI 2.520‐3.557) for IA with any formal help-seeking. In contrast, among adolescents without IA, help-seeking categories were generally associated with lower adjusted PRs. These findings indicate that adolescents with IA remained a high-prevalence group even when help-seeking was reported.

### Supplementary and Sensitivity Analyses

Supplementary analyses supported the robustness of the main findings. When depressive symptoms and anxiety symptoms were analyzed separately, the interaction patterns were generally consistent with the main analysis of mental health problems. In stratified models, informal-only help-seeking was associated with lower prevalence of depressive symptoms among adolescents with IA (PR 0.851, 95% CI 0.785‐0.922), whereas any formal help-seeking was not clearly associated with lower depressive symptoms (PR 0.905, 95% CI 0.771‐1.062). For anxiety symptoms among adolescents with IA, neither informal-only help-seeking (PR 0.882, 95% CI 0.772‐1.008) nor any formal help-seeking (PR 1.179, 95% CI 0.942‐1.474) showed a statistically clear lower-prevalence association (Table S7 in Multimedia Appendix 1). Observed outcome distributions across the 9 joint categories showed similarly high symptom burden among adolescents with IA, with mental health problem prevalence ranging from 58.6% to 67.6% across help-seeking categories (Table S8 in Multimedia Appendix 1).

Analyses using daily internet use duration as an alternative internet use measure showed a graded association with mental health outcomes. Compared with no internet use, the adjusted PRs for mental health problems were 1.328 (95% CI 1.242‐1.419) for less than 2 hours per day, 2.189 (95% CI 2.030‐2.361) for 2‐4 hours per day, and 3.289 (95% CI 3.023‐3.578) for 4 hours or more per day. Similar graded patterns were observed for depressive symptoms and anxiety symptoms (Table S9 in Multimedia Appendix 1). Continuous outcome models also showed that IA was associated with higher CESD-20 and GAD-7 scores, whereas informal-only and any formal help-seeking were associated with lower symptom scores in the overall sample (Table S10 in Multimedia Appendix 1).

Sensitivity analyses using alternative definitions of IA yielded consistent findings (Figure S1 in Multimedia Appendix 1). Continuous IA symptom scores were also positively associated with mental health problems. Each 1-point increase in the full 9-item IA symptom score was associated with a 33% higher prevalence of mental health problems (PR 1.330, 95% CI 1.317‐1.343). Similar associations were observed after removing item 9 (PR 1.339, 95% CI 1.325‐1.353) and after removing items 1, 2, and 9 (PR 1.442, 95% CI 1.420‐1.464), suggesting that the association was not driven solely by affect-laden items (Table S11 in Multimedia Appendix 1), although potential criterion overlap could not be fully ruled out. Single-year analyses for 2023 and 2024 produced results consistent with the pooled analysis (Figure S2 in Multimedia Appendix 1).

Additional help-seeking sensitivity analyses clarified the composition of the any formal help-seeking group. When help-seeking was reclassified into 4 mutually exclusive categories, informal-only help-seeking was associated with lower prevalence of mental health problems (PR 0.560, 95% CI 0.533‐0.589), formal-only help-seeking was associated with higher prevalence (PR 1.183, 95% CI 1.036‐1.351), and combined informal and formal help-seeking was associated with lower prevalence (PR 0.383, 95% CI 0.346‐0.424). Results were materially unchanged after excluding adolescents with formal-only help-seeking (Tables S12-S14 in Multimedia Appendix 1). These findings indicate that the main results for any formal help-seeking mainly reflected adolescents who had contact with formal services in combination with informal support, rather than an exclusively formal help-seeking pathway.

## Discussion

### Principal Findings

To our knowledge, this is the first large-scale population-based study to systematically examine the relationships among IA, mental health help-seeking behaviors, and mental health problems in adolescents. This study found that adolescents with IA had a markedly higher prevalence of depressive and anxiety symptoms and were also less likely to report mental health help-seeking, indicating a combined vulnerability of high mental health burden and low use of support. In the overall sample, informal-only and any formal help-seeking were associated with lower prevalence of mental health problems. However, stratified analyses showed that these inverse associations were weaker and less consistent among adolescents with IA, particularly for any formal help-seeking. The joint-category analysis further showed that adolescents with IA remained a high-prevalence group regardless of help-seeking category. Together, the results clarify how IA, help-seeking behaviors, and mental health problems are jointly patterned in adolescence. These findings also inform school-based and digital mental health strategies by identifying adolescents who may need low-threshold digital outreach, school-based digital screening, online help-seeking pathways, and better linkage between informal support and formal mental health services.

Lower levels of help-seeking among adolescents with IA may reflect intertwined psychological and social processes. Adolescents with IA may experience self-stigma, shame, emotional dysregulation, weakened offline social support, and avoidant coping, all of which can reduce perceived need, trust, and confidence in seeking help [36-44]. These findings suggest that interventions for IA should not focus solely on regulating internet use behaviors, but may also need to address help-seeking attitudes, stigma, mental health literacy, and social connectedness. Informal support from parents, peers, and teachers remains important because it is often more accessible and may serve as a bridge to professional services [17,25,45,46]. At the same time, formal services can provide assessment, guidance, and referral when needed. Therefore, school-based support systems should strengthen the linkage between informal support and formal mental health services rather than treating them as separate pathways.

This study found that 52.1% of junior middle school students reported ever seeking help from some source (formal or informal); yet, only 9.9% reported any formal psychological help-seeking (eg, counselors, hotlines, or hospitals). Among adolescents with IA, the gap was more pronounced: 59.9% reported no help-seeking, meaning that only 40.1% reported any help-seeking, and only 6.3% reported any formal help-seeking. Although direct comparisons with UK and US estimates are limited by differences in help-seeking definitions, reporting sources, and assessment time frames, broader international evidence suggests that adolescents’ use of professional mental health services remains limited [47-50]. Chinese studies similarly indicate that, among middle school students with notable depressive symptoms, only about 3.9%‐18% actively seek professional help [23,25,46]. In this context, the low formal help-seeking rate observed in this study, especially the 6.3% rate among adolescents with IA, suggests substantial service gaps among students with high psychological burden. This finding is consistent with current international and national policy efforts to expand adolescent mental health service coverage and strengthen school-based mental health service pathways [51-53]. Against this policy background, the low formal help-seeking rate among adolescents with IA highlights the need to strengthen the translation of school-based mental health resources into timely and accessible support for high-risk students.

Consistent with previous research linking adolescent internet use or IA with depressive symptoms and broader mental health problems [7,9,10], this study further confirmed the close association between IA and depressive and anxiety symptoms among adolescents. Problematic smartphone use, another form of digital behavioral difficulty, shows similar patterns. Studies have found that both newly developed and persistent smartphone dependence are associated with increased subsequent risks of depression and anxiety, while those whose dependence remitted no longer exhibited elevated risk [54]. Adolescents with IA are more likely to experience emotional dysregulation, social isolation, and poor sleep quality, which may contribute to elevated mental health problems [8,9,12,37]. Our results support this pattern in a large sample and further showed that informal-only and any formal help-seeking were associated with lower prevalence of mental health problems in the overall sample. However, stratified analyses indicated that these inverse associations were weaker and less consistent among adolescents with IA, particularly for any formal help-seeking, which was not clearly associated with lower prevalence of mental health problems within the IA group. These findings suggest that help-seeking is patterned differently across IA status and should not be interpreted as evidence that formal help-seeking provides stronger protection against IA-related mental health problems. Rather, they point to the need to understand why adolescents with IA remain a high-prevalence group even when help-seeking is reported, and whether the support they receive is timely, appropriate, and effectively linked to further care when needed. The 4-category sensitivity analysis further suggested that the overall association for any formal help-seeking mainly reflected adolescents who used formal services together with informal support, rather than an exclusively formal pathway. Outcome-specific supplementary analyses also showed that the relative pattern of informal-only and any formal help-seeking differed between depressive and anxiety symptoms; therefore, any formal help-seeking should not be interpreted as consistently showing a stronger inverse association than informal-only help-seeking. This pattern may reflect differences in symptom profiles, service needs, and pathways into care. Adolescents who use formal services may include those with more persistent or severe symptoms, whereas informal support may be more commonly used at earlier or less clinically severe stages of distress.

The findings of this study have important implications for adolescent mental health promotion. First, internet use has become an integral part of adolescents’ daily lives; thus, intervention strategies should shift from purely “risk control” to promoting “healthy use,” integrating behavioral guidance, mental health education, and digital safety protection. Second, multilayered psychological support networks across schools, families, and communities are needed to ensure that adolescents can access diverse and sustainable support resources when facing psychological distress. Third, particular attention should be paid to the “help-seeking gap” among adolescents at risk of IA. These findings are particularly relevant to the development of digital mental health strategies for adolescents. Adolescents with IA who do not seek help may represent a priority group for low-threshold digital outreach because they combine a high prevalence of mental health problems with limited use of support resources. School-based digital screening systems could incorporate indicators of internet use duration, IA symptoms, depressive symptoms, anxiety symptoms, and help-seeking status to identify students who may need timely follow-up. Online help-seeking platforms may also serve as an entry point for adolescents who are reluctant to seek face-to-face support because of stigma, low mental health literacy, or uncertainty about available services. Decision-aid tools may further support adolescents’ disclosure and help-seeking decisions by reducing cognitive and emotional uncertainty during the help-seeking process [55]. Importantly, such platforms should be embedded within a stepped-care pathway that connects anonymous psychoeducation and self-screening with peer or teacher support, school counseling, psychological hotlines, and referral to medical services when needed. Systematic reviews indicate that internet-based and digital mental health resources can complement offline help-seeking by offering additional channels for adolescents who prefer anonymity or self-reliance, while also highlighting the need to ensure resource credibility, privacy protection, and appropriate linkage to professional support in program design [56].

This study has several strengths. First, it used a large, provincially representative dataset with rigorous quality control, providing a strong basis for the generalizability of findings within Shandong Province. Second, by using Poisson regression with school-clustered CR2 robust SEs and conducting stratified and joint-category analyses, the findings were presented using PRs that are more interpretable for common outcomes. Third, the study simultaneously examined IA, help-seeking behaviors, and mental health outcomes, showing how the association between help-seeking and mental health problems differed across IA status.

Nonetheless, some limitations should be acknowledged. First, because of its cross-sectional design, this study cannot establish temporal or causal relationships among IA, help-seeking behaviors, and mental health problems. Mental health problems may increase internet use or reduce help-seeking, and adolescents with less severe symptoms may be more able or willing to seek help, whereas those with more severe distress may experience helplessness, stigma, shame, or emotional exhaustion that discourages help-seeking. Therefore, the observed associations should not be interpreted as evidence that help-seeking reduces or prevents mental health problems. Second, the study used repeated anonymous surveillance data from 2023 and 2024. Because some monitoring schools were retained across years and individual students could not be linked, repeated participation by the same students could not be identified. The pooled analysis therefore assumed individual-level independence. However, single-year sensitivity analyses yielded generally consistent results, suggesting that the main findings were unlikely to be driven solely by potential repeated participation across waves. Third, IA, help-seeking behaviors, and mental health outcomes were self-reported, which may have introduced recall bias, social desirability bias, or underreporting, especially for potentially sensitive behaviors and experiences such as addictive internet use, psychological distress, and mental health service use. Fourth, IA was measured using items adapted from the *DSM-5* symptom framework for Internet Gaming Disorder. Because Internet Gaming Disorder is specific to gaming, whereas our items referred to general internet use across devices and online activities, this measure should not be interpreted as assessing Internet Gaming Disorder or providing a clinical diagnosis. Some *DSM-5*–derived criteria, such as preoccupation, tolerance, withdrawal-like symptoms, and using the internet to relieve negative mood, may not fully distinguish intensive engagement from problematic involvement and may overlap with emotional symptoms. The dual threshold of at least 4 hours per day and at least 4 symptoms followed a previous Chinese adolescent surveillance study [28], but no external clinical diagnostic interview was available to estimate its sensitivity or specificity. Therefore, the observed IA prevalence of 1.4% should be interpreted as the prevalence of a relatively severe operational IA phenotype rather than the full spectrum of problematic internet use. Future studies should use validated diagnostic instruments, longer assessment windows, clinical interviews, objective digital-use records, and content-specific measures that distinguish gaming, social media, short-video use, and other online activities. Fifth, the help-seeking variable asked whether students had ever sought help for psychological problems, without specifying whether help-seeking was recent, ongoing, or occurred much earlier. Therefore, this variable should be interpreted as a history of help-seeking rather than current or recent support. This difference in assessment windows should be considered when interpreting the inverse associations between help-seeking history and current depressive or anxiety symptoms. Sixth, the any formal help-seeking category included students who reported school counseling, a psychological hotline, or hospital services, regardless of whether they also reported informal sources. Thus, this category was not equivalent to formal-only help-seeking. We addressed this classification issue through sensitivity analyses using 4 mutually exclusive help-seeking categories and by excluding formal-only help-seeking, but future studies should collect more detailed information on the sequence, intensity, duration, and perceived quality of informal and formal support. Seventh, residual confounding remains possible. The study did not include several important family-, peer-, school-, and treatment-related factors, such as family socioeconomic status, parental mental health, family functioning or conflict, peer relationships, academic performance or pressure, and prior mental health treatment history. These factors may influence IA, help-seeking behaviors, and mental health problems simultaneously. For example, family conflict or low social support may increase problematic internet use, discourage help-seeking, and worsen mental health, which could bias the association between IA and mental health problems, potentially away from the null [17,46,57,58]. Conversely, higher family resources, better mental health literacy, and more supportive interpersonal environments may increase help-seeking while also being associated with better mental health, potentially exaggerating the inverse association between help-seeking and mental health problems [17,45,59,60]. For formal help-seeking, prior treatment history or more severe preexisting symptoms may also influence selection into formal services, which could attenuate or reverse the observed association [45]. Future studies should collect richer family, school, peer, academic, and treatment-history information and adopt longitudinal designs to better address residual confounding and clarify the temporal sequence linking IA, help-seeking behaviors, and mental health problems.

### Conclusion

This repeated cross-sectional study found that IA was strongly associated with a higher prevalence of depressive and anxiety symptoms among Chinese adolescents and was also associated with lower levels of mental health help-seeking. In the overall sample, informal-only and any formal help-seeking were associated with lower prevalence of mental health problems; however, these inverse associations were weaker and less consistent among adolescents with IA, particularly for any formal help-seeking. These findings highlight a high-burden, low help-seeking subgroup of adolescents with IA and suggest the need to improve timely access to appropriate support. Strengthening school-based mental health services, improving linkage between informal and formal support, reducing stigma around help-seeking, and developing low-threshold digital mental health pathways may help better connect adolescents with IA to needed mental health resources. In particular, school-based digital screening, anonymous online help-seeking entry points, and blended digital-offline referral systems may be useful components of future adolescent mental health support strategies.

## Supplementary material

10.2196/90014 Multimedia Appendix 1 Supplementary methods, figures, and tables for the measurement of internet addiction, additional analyses, and sensitivity analyses.

10.2196/90014 Checklist 1 STROBE checklist.

## Abbreviations

CESD-20: 20-item Center for Epidemiologic Studies Depression Scale
*DSM-5*: *Diagnostic and Statistical Manual of Mental Disorders* (Fifth Edition)
GAD-7: 7-item Generalized Anxiety Disorder Scale
IA: internet addiction
OR: odds ratio
PR: prevalence ratio
STROBE: Strengthening the Reporting of Observational Studies in Epidemiology
