# Supplementary material for: Internet Addiction, Mental Health Help-Seeking, and Mental Health Problems Among Chinese Adolescents: Repeated Cross-Sectional Study
Source: J Med Internet Res. 2026 Aug 12;28:e90014. doi: 10.2196/90014 (PMC13466870; doi:10.2196/90014)
Supplement: Supplementary file 1 [file jmir-v28-e90014-s001.docx]

**Multimedia Appendix 1**

**Contents**

[Methods S1. Measurement of Internet Addiction.	2](#_Toc30522)

[Methods S2. Supplementary analyses	3](#_Toc14851)

[Methods S3. Sensitivity analyses	3](#_Toc3528)

[Figure S1. Forest plot of sensitivity analyses using alternative definitions of internet addiction in relation to mental health outcomes	5](#_Toc14541)

[Figure S2. Single-year sensitivity analyses of associations between internet addiction status, help-seeking, and mental health outcomes	6](#_Toc5299)

[Table S1. Distribution of individual help-seeking sources	7](#_Toc14925)

[Table S2. Associations of internet addiction status and mental health help-seeking with depressive symptoms	8](#_Toc27912)

[Table S3. Interaction between internet addiction status and mental health help-seeking on depressive symptoms	9](#_Toc15343)

[Table S4. Associations of internet addiction status and mental health help-seeking with anxiety symptoms	10](#_Toc25833)

[Table S5. Interaction between internet addiction status and mental health help-seeking on anxiety symptoms	11](#_Toc2080)

[Table S6. Secondary logistic regression analysis of the interaction between internet addiction status and mental health help-seeking on mental health problems	12](#_Toc26233)

[Table S7. Stratified associations of help-seeking with depressive and anxiety symptoms within each internet addiction status	13](#_Toc19491)

[Table S8. Observed prevalence of mental health outcomes and symptom scores according to internet addiction status and help-seeking category	14](#_Toc20214)

[Table S9. Associations of daily internet use duration and mental health help-seeking with mental health outcomes	15](#_Toc18744)

[Table S10. Associations of internet addiction status and mental health help-seeking with continuous depressive and anxiety symptom scores	16](#_Toc14258)

[Table S11. Sensitivity analyses using continuous internet addiction symptom scores	17](#_Toc25652)

[Table S12. Sensitivity analysis using four-category help-seeking classification	18](#_Toc2000)

[Table S13. Sensitivity analysis using four-category help-seeking classification for mental health problems	19](#_Toc11975)

[Table S14. Sensitivity analysis excluding adolescents with formal-only help-seeking for mental health problems	21](#_Toc7406)

# Methods S1. Measurement of Internet Addiction.

Internet Usage (Internet use includes mobile phones, tablets, computers, etc.)

a Have you never used the internet?

Options: Yes; No

b In the past 7 days, what was your average daily internet usage time?______ hours per day

c Do you experience the following situations?

1. Frequently use the internet, and even when not online, thoughts related to the internet persistently occupy your mind.

Options: Yes; No

2. Feel uncomfortable or reluctant to engage in other activities when unable to access the internet, with these feelings being alleviated upon going online.

Options: Yes; No

3. Need to increase internet usage time to achieve satisfaction.

Options: Yes; No

4. Have lost interest in other recreational activities (e.g., hobbies, meeting friends) due to internet use.

Options: Yes; No

5. Have repeatedly attempted to reduce or stop internet use but are unable to control it.

Options: Yes; No

6. Have failed to complete schoolwork or have skipped school because of internet use.

Options: Yes; No

7. Have concealed the extent of your internet use from parents, teachers, or classmates.

Options: Yes; No

8. Continue using the internet despite knowing the negative consequences (e.g., insufficient sleep, being late for class, arguments with parents).

Options: Yes; No

9. Use the internet to escape from reality, or to relieve feelings of helplessness, anxiety, or depression.

Options: Yes; No

# Methods S2. Supplementary analyses

Several supplementary analyses were conducted to examine whether the main findings were consistent across different outcome specifications and internet-use measures. First, depressive symptoms and anxiety symptoms were analyzed separately as binary outcomes to determine whether the associations observed for the composite mental health problem outcome were also present for each symptom domain. For these two outcomes, we fitted the same Poisson regression models with a log link and school-clustered CR2 robust standard errors as in the main analyses. The models included main-effect models for internet addiction status and help-seeking category, multiplicative interaction models between internet addiction status and help-seeking category, and help-seeking models stratified by internet addiction status. The reference categories were non-addictive internet use for internet addiction status and no help-seeking for help-seeking category. Second, to provide an unadjusted description of outcome distributions, we tabulated the observed prevalence of mental health problems, depressive symptoms, and anxiety symptoms, as well as mean CESD-20 and GAD-7 scores, across the nine joint categories formed by internet addiction status and help-seeking category. Third, daily internet use duration was analyzed as an alternative exposure to assess whether the association between internet use and mental health problems showed a graded pattern independent of the internet addiction classification. Daily internet use duration was categorized as no internet use, less than 2 hours per day, 2 to 4 hours per day, and 4 hours or more per day. Poisson regression models were fitted for mental health problems, depressive symptoms, and anxiety symptoms, with no internet use as the reference category for duration and no help-seeking as the reference category for help-seeking. These models adjusted for the same covariates as the main analyses, including survey year, sex, age, grade, monitoring site, ethnicity, boarding status, chronic disease history, myopia status, and BMI category. Finally, depressive and anxiety symptom scores were analyzed as continuous secondary outcomes. CESD-20 and GAD-7 scores were modeled using linear regression with school-clustered CR2 robust standard errors, with internet addiction status, help-seeking category, and the full covariate set included in the models. Regression coefficients and 95% confidence intervals were reported to indicate adjusted differences in symptom scores.

# Methods S3. Sensitivity analyses

Several sensitivity analyses were performed to evaluate the robustness of the findings to alternative exposure definitions, repeated cross-sectional sampling, and the classification of help-seeking sources. To address uncertainty in the operational definition of internet addiction, we constructed a series of alternative binary definitions using different combinations of daily internet use duration and symptom thresholds. These included the definition of at least 4 hours per day and at least 5 endorsed symptoms, a symptom-only definition using at least 5 endorsed symptoms without a time threshold, a broader duration threshold of at least 2 hours per day combined with at least 5 endorsed symptoms, broader symptom thresholds of at least 3 or at least 1 endorsed symptom combined with at least 4 hours per day, a definition excluding item 9, and a definition excluding items 1, 2, and 9. The latter two analyses were conducted to examine whether the findings were influenced by potential overlap between internet addiction items and emotional symptoms. Each alternative internet addiction definition was entered separately into Poisson regression models for mental health problems, depressive symptoms, and anxiety symptoms, with adjustment for the full covariate set and school-clustered CR2 robust standard errors. In addition, continuous internet addiction symptom scores were analyzed, including the full 9-item score, the score excluding item 9, and the score excluding items 1, 2, and 9; these models estimated the adjusted prevalence ratio associated with each 1-point increase in the corresponding symptom score. To assess the potential influence of the repeated cross-sectional design, single-year sensitivity analyses were conducted separately for the 2023 and 2024 survey waves. These models used the same Poisson regression framework and covariate adjustment strategy as the main models, except that survey year was omitted from the covariate set. This analysis was conducted because the monitoring schools were generally consistent across years, whereas the anonymous survey design did not allow repeated individual participation to be identified. Finally, two help-seeking sensitivity analyses were conducted to examine the implications of overlap between informal and formal help-seeking sources. First, help-seeking was reclassified into four mutually exclusive categories: no help-seeking, informal-only help-seeking, formal-only help-seeking, and both informal and formal help-seeking. Using this four-category variable, we fitted main-effect models, interaction models between internet addiction status and help-seeking category, and internet-addiction-stratified help-seeking models for mental health problems. Second, adolescents with formal-only help-seeking were excluded, and the models were re-estimated using three categories: no help-seeking, informal-only help-seeking, and both informal and formal help-seeking. In these help-seeking sensitivity analyses, non-addictive internet use and no help-seeking remained the reference categories in the main-effect and interaction models, and no help-seeking was used as the reference category within each internet addiction stratum.

# Figure S1. Forest plot of sensitivity analyses using alternative definitions of internet addiction in relation to mental health outcomes

Notes:

IA = internet addiction; PR = prevalence ratio; CI = confidence interval.

Points and horizontal lines represent adjusted PRs and 95% CIs from Poisson regression models with school-clustered CR2 robust standard errors. Models were adjusted for survey year, sex, age, grade, monitoring site, ethnicity, boarding status, chronic disease history, myopia status, and BMI category. Mental health problems were defined as clinically significant depressive symptoms or anxiety symptoms. Depressive symptoms were defined as CESD-20 ≥16, and anxiety symptoms were defined as GAD-7 ≥10. Item 9 refers to using the internet to escape reality or relieve negative emotions; items 1 and 2 refer to preoccupation/withdrawal-related symptoms.

# Figure S2. Single-year sensitivity analyses of associations between internet addiction status, help-seeking, and mental health outcomes

Notes:

PR = prevalence ratio; CI = confidence interval.

Mental health problems were defined as clinically significant depressive symptoms or anxiety symptoms. Depressive symptoms were defined as CESD-20 ≥16, and anxiety symptoms were defined as GAD-7 ≥10.

Poisson regression models with school-clustered CR2 robust standard errors were used. Models adjusted for sex, age, grade, monitoring site, ethnicity, boarding status, chronic disease history, myopia status, and BMI category. Survey year was not included as a covariate in the single-year models.

The reference group for internet addiction status was non-addictive internet use, and the reference group for help-seeking was no help-seeking.

# Table S1. Distribution of individual help-seeking sources.

| Help-seeking source | n (% of total sample) | n (% among help-seekers) |
| --- | --- | --- |
| Parent | 74,413 (43.27%) | 74,413 (83.08%) |
| Teacher | 32,983 (19.18%) | 32,983 (36.82%) |
| Classmates/friends | 45,741 (26.60%) | 45,741 (51.07%) |
| Online sources | 10,223 (5.94%) | 10,223 (11.41%) |
| School counseling office | 12,358 (7.19%) | 12,358 (13.80%) |
| Psychological hotline | 7,721 (4.49%) | 7,721 (8.62%) |
| Hospital services | 8,770 (5.10%) | 8,770 (9.79%) |

Notes:

Percentages of the total sample were calculated using all participants as the denominator. Percentages among help-seekers were calculated using participants who reported any mental health help-seeking as the denominator. Participants could report more than one help-seeking source, so percentages do not sum to 100%.

# Table S2. Associations of internet addiction status and mental health help-seeking with depressive symptoms.

| Variables | PR (95% CI) | P value | OR (95% CI) | P value |
| --- | --- | --- | --- | --- |
| Internet addiction status |  |  |  |  |
| Non-addictive Internet use | 1 (Ref.) |  | 1 (Ref.) |  |
| No Internet use | 0.620 (0.580–0.663) | <0.001 | 0.574 (0.532–0.619) | <0.001 |
| Internet addiction | 3.714 (3.475–3.969) | <0.001 | 9.134 (7.835–10.648) | <0.001 |
| Mental health help-seeking |  |  |  |  |
| No help-seeking | 1 (Ref.) |  | 1 (Ref.) |  |
| Informal-only help-seeking | 0.576 (0.544–0.609) | <0.001 | 0.509 (0.476–0.545) | <0.001 |
| Any formal help-seeking | 0.456 (0.415–0.501) | <0.001 | 0.393 (0.353–0.436) | <0.001 |

Notes: PR = prevalence ratio; OR = odds ratio; CI = confidence interval. Depressive symptoms were defined as CESD-20 ≥16. PRs were estimated using Poisson regression with school-clustered CR2 robust standard errors. ORs were estimated using logistic regression with school-clustered CR2 robust standard errors. Models adjusted for survey year, sex, age, grade, monitoring site, ethnicity, boarding status, chronic disease history, myopia status, and BMI category.

# Table S3. Interaction between internet addiction status and mental health help-seeking on depressive symptoms.

| Variables | PR (95% CI) | P value |
| --- | --- | --- |
| Internet addiction status |  |  |
| Non-addictive Internet use | 1 (Ref.) |  |
| No Internet use | 0.658 (0.601–0.721) | <0.001 |
| Internet addiction | 3.227 (2.991–3.481) | <0.001 |
| Mental health help-seeking |  |  |
| No help-seeking | 1 (Ref.) |  |
| Informal-only help-seeking | 0.578 (0.543–0.614) | <0.001 |
| Any formal help-seeking | 0.460 (0.417–0.508) | <0.001 |
| Internet addiction status × Mental health help-seeking |  |  |
| Non-addictive Internet use × No help-seeking | 1 (Ref.) |  |
| No Internet use × Informal-only help-seeking | 0.858 (0.754–0.976) | 0.021 |
| Internet addiction × Informal-only help-seeking | 1.490 (1.349–1.645) | <0.001 |
| No Internet use × Any formal help-seeking | 0.777 (0.599–1.007) | 0.059 |
| Internet addiction × Any formal help-seeking | 1.962 (1.626–2.366) | <0.001 |

Notes:

PR = prevalence ratio; CI = confidence interval.

Depressive symptoms were defined as CESD-20 ≥16.

Models include school ID as a random intercept and adjust for all covariates used in the main analyses.

p-values adjusted using the BH procedure.

# Table S4. Associations of internet addiction status and mental health help-seeking with anxiety symptoms.

| Variables | PR (95% CI) | P value | OR (95% CI) | P value |
| --- | --- | --- | --- | --- |
| Internet addiction status |  |  |  |  |
| Non-addictive Internet use | 1 (Ref.) |  | 1 (Ref.) |  |
| No Internet use | 0.571 (0.507–0.643) | <0.001 | 0.555 (0.490–0.629) | <0.001 |
| Internet addiction | 5.546 (4.963–6.197) | <0.001 | 8.151 (7.109–9.346) | <0.001 |
| Mental health help-seeking |  |  |  |  |
| No help-seeking | 1 (Ref.) |  | 1 (Ref.) |  |
| Informal-only help-seeking | 0.543 (0.489–0.603) | <0.001 | 0.516 (0.461–0.579) | <0.001 |
| Any formal help-seeking | 0.583 (0.512–0.663) | <0.001 | 0.558 (0.486–0.641) | <0.001 |

Notes: PR = prevalence ratio; OR = odds ratio; CI = confidence interval. Anxiety symptoms were defined as GAD-7 ≥10. PRs were estimated using Poisson regression with school-clustered CR2 robust standard errors. ORs were estimated using logistic regression with school-clustered CR2 robust standard errors. Models adjusted for survey year, sex, age, grade, monitoring site, ethnicity, boarding status, chronic disease history, myopia status, and BMI category.

# Table S5. Interaction between internet addiction status and mental health help-seeking on anxiety symptoms.

| Variables | PR (95% CI) | P value |
| --- | --- | --- |
| Internet addiction status |  |  |
| Non-addictive Internet use | 1 (Ref.) |  |
| No Internet use | 0.646 (0.544–0.766) | <0.001 |
| Internet addiction | 4.686 (4.072–5.393) | <0.001 |
| Mental health help-seeking |  |  |
| No help-seeking | 1 (Ref.) |  |
| Informal-only help-seeking | 0.548 (0.481–0.624) | <0.001 |
| Any formal help-seeking | 0.601 (0.514–0.702) | <0.001 |
| Internet addiction status × Mental health help-seeking |  |  |
| Non-addictive Internet use × No help-seeking | 1 (Ref.) |  |
| No Internet use × Informal-only help-seeking | 0.723 (0.580–0.901) | 0.004 |
| Internet addiction × Informal-only help-seeking | 1.620 (1.356–1.936) | <0.001 |
| No Internet use × Any formal help-seeking | 0.572 (0.416–0.788) | <0.001 |
| Internet addiction × Any formal help-seeking | 1.948 (1.462–2.596) | <0.001 |

Notes: PR = prevalence ratio; CI = confidence interval. Anxiety symptoms were defined as GAD-7 ≥10. Models used Poisson regression with school-clustered CR2 robust standard errors and adjusted for survey year, sex, age, grade, monitoring site, ethnicity, boarding status, chronic disease history, myopia status, and BMI category. The reference group for internet addiction status was non-addictive internet use, and the reference group for help-seeking was no help-seeking.

# Table S6. Secondary logistic regression analysis of the interaction between internet addiction status and mental health help-seeking on mental health problems.

| Variables | OR (95% CI) | P value |
| --- | --- | --- |
| **Internet addiction status** |  |  |
| Non-addictive Internet use | 1 (Ref.) |  |
| No Internet use | 0.607 (0.547–0.675) | <0.001 |
| Internet addiction | 8.060 (6.595–9.851) | <0.001 |
| **Mental health help-seeking** |  |  |
| No help-seeking | 1 (Ref.) |  |
| Informal-only help-seeking | 0.501 (0.469–0.535) | <0.001 |
| Any formal help-seeking | 0.402 (0.360–0.449) | <0.001 |
| **Internet addiction status × Mental health help-seeking** |  |  |
| Non-addictive Internet use × No help-seeking | 1 (Ref.) |  |
| No Internet use × Informal-only help-seeking | 0.868 (0.749–1.005) | 0.060 |
| Internet addiction × Informal-only help-seeking | 1.297 (1.029–1.636) | 0.030 |
| No Internet use × Any formal help-seeking | 0.826 (0.629–1.084) | 0.170 |
| Internet addiction × Any formal help-seeking | 2.166 (1.351–3.471) | 0.002 |

Notes:

OR = odds ratio; CI = confidence interval.

These logistic regression estimates are presented as secondary analyses only. The primary estimates are adjusted prevalence ratios from Poisson regression models, as reported in the main text Table 4. Models used school-clustered CR2 robust standard errors and adjusted for survey year, sex, age, grade, monitoring site, ethnicity, boarding status, chronic disease history, myopia status, and BMI category.

# Table S7. Stratified associations of help-seeking with depressive and anxiety symptoms within each internet addiction status.

| Internet addiction status | Help-seeking category | Depressive symptoms, PR (95% CI) | P value | Anxiety symptoms, PR (95% CI) | P value |
| --- | --- | --- | --- | --- | --- |
| Non-addictive Internet use | No help-seeking | 1 (Ref.) |  | 1 (Ref.) |  |
|  | Informal-only help-seeking | 0.577 (0.543–0.613) | <0.001 | 0.547 (0.481–0.622) | <0.001 |
|  | Any formal help-seeking | 0.460 (0.417–0.508) | <0.001 | 0.603 (0.516–0.703) | <0.001 |
| No Internet use | No help-seeking | 1 (Ref.) |  | 1 (Ref.) |  |
|  | Informal-only help-seeking | 0.496 (0.434–0.566) | <0.001 | 0.398 (0.332–0.476) | <0.001 |
|  | Any formal help-seeking | 0.358 (0.278–0.460) | <0.001 | 0.343 (0.259–0.454) | <0.001 |
| Internet addiction | No help-seeking | 1 (Ref.) |  | 1 (Ref.) |  |
|  | Informal-only help-seeking | 0.851 (0.785–0.922) | <0.001 | 0.882 (0.772–1.008) | 0.067 |
|  | Any formal help-seeking | 0.905 (0.771–1.062) | 0.224 | 1.179 (0.942–1.474) | 0.154 |

Notes:

PR = prevalence ratio; CI = confidence interval.

Depressive symptoms were defined as CESD-20 ≥16, and anxiety symptoms were defined as GAD-7 ≥10.

Poisson regression models with school-clustered CR2 robust standard errors were used. Models adjusted for survey year, sex, age, grade, monitoring site, ethnicity, boarding status, chronic disease history, myopia status, and BMI category.

Within each internet addiction status, no help-seeking was used as the reference group.

# Table S8. Observed prevalence of mental health outcomes and symptom scores according to internet addiction status and help-seeking category.

| Internet addiction status | Help-seeking category | N (%) | Mental health problems, n (%) | Depressive symptoms, n (%) | Anxiety symptoms, n (%) | CESD-20 score, mean | GAD-7 score, mean |
| --- | --- | --- | --- | --- | --- | --- | --- |
| Non-addictive Internet use | No help-seeking | 58,040 (47.43) | 11,965 (20.62) | 11,556 (19.91) | 4,077 (7.02) | 10.28 | 3.00 |
|  | Informal-only help-seeking | 52,325 (42.76) | 6,415 (12.26) | 6,129 (11.71) | 2,051 (3.92) | 8.17 | 2.23 |
|  | Any formal help-seeking | 12,003 (9.81) | 1,144 (9.53) | 1,099 (9.16) | 505 (4.21) | 7.26 | 1.72 |
| No Internet use | No help-seeking | 22,922 (48.57) | 3,041 (13.27) | 2,913 (12.71) | 1,006 (4.39) | 8.97 | 1.99 |
|  | Informal-only help-seeking | 19,322 (40.94) | 1,295 (6.70) | 1,228 (6.36) | 339 (1.75) | 7.12 | 1.30 |
|  | Any formal help-seeking | 4,952 (10.49) | 242 (4.89) | 226 (4.56) | 75 (1.51) | 6.29 | 0.86 |
| Internet addiction | No help-seeking | 1,440 (59.85) | 974 (67.64) | 953 (66.18) | 482 (33.47) | 23.16 | 8.04 |
|  | Informal-only help-seeking | 814 (33.83) | 477 (58.60) | 468 (57.49) | 245 (30.10) | 21.56 | 7.03 |
|  | Any formal help-seeking | 152 (6.32) | 99 (65.13) | 93 (61.18) | 61 (40.13) | 25.70 | 8.68 |

Notes:

Values are presented as n (%) for categorical outcomes and mean scores for continuous outcomes. Mental health problems were defined as the presence of clinically significant depressive symptoms (CESD-20 ≥16) or anxiety symptoms (GAD-7 ≥10). This table presents observed (unadjusted) outcome distributions and symptom scores according to the joint categories of internet addiction status and mental health help-seeking.

# Table S9. Associations of daily internet use duration and mental health help-seeking with mental health outcomes.

| Variables | Mental health problems, PR (95% CI) | P value | Depressive symptoms, PR (95% CI) | P value | Anxiety symptoms, PR (95% CI) | P value |
| --- | --- | --- | --- | --- | --- | --- |
| Daily internet use duration |  |  |  |  |  |  |
| No Internet use | 1 (Ref.) |  | 1 (Ref.) |  | 1 (Ref.) |  |
| <2 h/day | 1.328 (1.242–1.419) | <0.001 | 1.335 (1.247–1.429) | <0.001 | 1.448 (1.288–1.627) | <0.001 |
| 2–4 h/day | 2.189 (2.030–2.361) | <0.001 | 2.214 (2.049–2.392) | <0.001 | 2.367 (2.099–2.669) | <0.001 |
| ≥4 h/day | 3.289 (3.023–3.578) | <0.001 | 3.340 (3.063–3.642) | <0.001 | 4.441 (3.760–5.245) | <0.001 |
| Mental health help-seeking |  |  |  |  |  |  |
| No help-seeking | 1 (Ref.) |  | 1 (Ref.) |  | 1 (Ref.) |  |
| Informal-only help-seeking | 0.595 (0.565–0.627) | <0.001 | 0.590 (0.559–0.622) | <0.001 | 0.556 (0.504–0.612) | <0.001 |
| Any formal help-seeking | 0.468 (0.428–0.511) | <0.001 | 0.463 (0.423–0.507) | <0.001 | 0.589 (0.521–0.666) | <0.001 |

Notes:

PR = prevalence ratio; CI = confidence interval.

Mental health problems were defined as clinically significant depressive symptoms or anxiety symptoms. Depressive symptoms were defined as CESD-20 ≥16, and anxiety symptoms were defined as GAD-7 ≥10.

Poisson regression models with school-clustered CR2 robust standard errors were used. Models adjusted for survey year, sex, age, grade, monitoring site, ethnicity, boarding status, chronic disease history, myopia status, and BMI category.

The reference group for daily internet use duration was no Internet use, and the reference group for help-seeking was no help-seeking.

# Table S10. Associations of internet addiction status and mental health help-seeking with continuous depressive and anxiety symptom scores.

| Variables | CESD-20 score, β(95% CI) | P value | GAD-7 score, β(95% CI) | P value |
| --- | --- | --- | --- | --- |
| Internet addiction status |  |  |  |  |
| Non-addictive Internet use | 1 (Ref.) |  | 1 (Ref.) |  |
| No Internet use | -1.042 (-1.255 to -0.829) | <0.001 | -0.900 (-0.980 to -0.820) | <0.001 |
| Internet addiction | 13.175 (12.340 to 14.011) | <0.001 | 5.046 (4.626 to 5.467) | <0.001 |
| Mental health help-seeking |  |  |  |  |
| No help-seeking | 1 (Ref.) |  | 1 (Ref.) |  |
| Informal-only help-seeking | -2.051 (-2.247 to -1.855) | <0.001 | -0.782 (-0.872 to -0.693) | <0.001 |
| Any formal help-seeking | -2.868 (-3.206 to -2.530) | <0.001 | -1.213 (-1.329 to -1.097) | <0.001 |

Notes:

β = regression coefficient; CI = confidence interval. CESD-20 and GAD-7 scores were analyzed as continuous outcomes. Linear regression models with school-clustered CR2 robust standard errors were used. Models adjusted for survey year, sex, age, grade, monitoring site, ethnicity, boarding status, chronic disease history, myopia status, and BMI category. The reference group for internet addiction status was non-addictive internet use, and the reference group for help-seeking was no help-seeking.

# Table S11. Sensitivity analyses using continuous internet addiction symptom scores.

| IA symptom score | PR (95% CI) | P value |
| --- | --- | --- |
| IA score based on all 9 items | 1.330 (1.317–1.343) | <0.001 |
| IA score after removing item 9 | 1.339 (1.325–1.353) | <0.001 |
| IA score after removing items 1, 2, and 9 | 1.442 (1.420–1.464) | <0.001 |

Notes:

IA = internet addiction; PR = prevalence ratio; CI = confidence interval.

The outcome was mental health problems, defined as clinically significant depressive symptoms or anxiety symptoms.

Poisson regression models with school-clustered CR2 robust standard errors were used. Models adjusted for survey year, sex, age, grade, monitoring site, ethnicity, boarding status, chronic disease history, myopia status, and BMI category.

PRs represent the association with each 1-point increase in the corresponding IA symptom score. Item 9 refers to using the internet to escape reality or relieve helplessness, anxiety, or depressive feelings. Items 1 and 2 refer to preoccupation/withdrawal-related symptoms.

# Table S12. Sensitivity analysis using four-category help-seeking classification.

| Variables | n (%) | PR (95% CI) | P value |
| --- | --- | --- | --- |
| Internet addiction status |  |  |  |
| Non-addictive Internet use | 122,368 (71.16) | 1 (Ref.) |  |
| No Internet use | 47,196 (27.44) | 0.624 (0.584–0.667) | <0.001 |
| Internet addiction | 2,406 (1.40) | 3.620 (3.386–3.869) | <0.001 |
| Four-category help-seeking classification |  |  |  |
| No help-seeking | 82,402 (47.92) | 1 (Ref.) |  |
| Informal only | 72,461 (42.14) | 0.560 (0.533–0.589) | <0.001 |
| Formal only | 1,548 (0.90) | 1.183 (1.036–1.351) | 0.015 |
| Both informal and formal | 15,559 (9.05) | 0.383 (0.346–0.424) | <0.001 |

Notes:

PR = prevalence ratio; CI = confidence interval.

The outcome was mental health problems, defined as clinically significant depressive symptoms or anxiety symptoms.

Poisson regression models with school-clustered CR2 robust standard errors were used. Models adjusted for survey year, sex, age, grade, monitoring site, ethnicity, boarding status, chronic disease history, myopia status, and BMI category.

The four-category help-seeking variable separated no help-seeking, informal-only help-seeking, formal-only help-seeking, and combined informal and formal help-seeking.

Because the formal-only group was small, this analysis was considered exploratory.

# Table S13. Sensitivity analysis using four-category help-seeking classification for mental health problems.

| Model | IA status | Variable/comparison | Adjusted PR (95% CI) | P value |
| --- | --- | --- | --- | --- |
| Main-effect model | Overall | No Internet use vs non-addictive internet use | 0.624 (0.584-0.667) | <0.001 |
| Main-effect model | Overall | Internet addiction vs non-addictive internet use | 3.620 (3.386-3.869) | <0.001 |
| Main-effect model | Overall | Informal only vs no help-seeking | 0.560 (0.533-0.589) | <0.001 |
| Main-effect model | Overall | Formal only vs no help-seeking | 1.183 (1.036-1.351) | 0.015 |
| Main-effect model | Overall | Both informal and formal vs no help-seeking | 0.383 (0.346-0.424) | <0.001 |
| Interaction model | Overall | No Internet use vs non-addictive internet use | 0.664 (0.607-0.726) | <0.001 |
| Interaction model | Overall | Internet addiction vs non-addictive internet use | 3.171 (2.942-3.417) | <0.001 |
| Interaction model | Overall | Informal only vs no help-seeking | 0.564 (0.533-0.596) | <0.001 |
| Interaction model | Overall | Formal only vs no help-seeking | 1.247 (1.069-1.455) | 0.006 |
| Interaction model | Overall | Both informal and formal vs no help-seeking | 0.382 (0.344-0.425) | <0.001 |
| Interaction model | Overall | No Internet use × Informal only | 0.840 (0.739-0.956) | 0.009 |
| Interaction model | Overall | Internet addiction × Informal only | 1.510 (1.371-1.662) | <0.001 |
| Interaction model | Overall | No Internet use × Formal only | 0.734 (0.544-0.991) | 0.047 |
| Interaction model | Overall | Internet addiction × Formal only | 1.009 (0.791-1.287) | 0.943 |
| Interaction model | Overall | No Internet use × Both informal and formal | 0.823 (0.615-1.103) | 0.195 |
| Interaction model | Overall | Internet addiction × Both informal and formal | 2.178 (1.730-2.743) | <0.001 |
| IA-stratified help-seeking model | Non-addictive Internet use | Informal only vs no help-seeking | 0.563 (0.533-0.594) | <0.001 |
| IA-stratified help-seeking model | Non-addictive Internet use | Formal only vs no help-seeking | 1.243 (1.066-1.450) | 0.007 |
| IA-stratified help-seeking model | Non-addictive Internet use | Both informal and formal vs no help-seeking | 0.383 (0.345-0.425) | <0.001 |
| IA-stratified help-seeking model | No Internet use | Informal only vs no help-seeking | 0.475 (0.419-0.539) | <0.001 |
| IA-stratified help-seeking model | No Internet use | Formal only vs no help-seeking | 0.927 (0.692-1.243) | 0.615 |
| IA-stratified help-seeking model | No Internet use | Both informal and formal vs no help-seeking | 0.315 (0.238-0.418) | <0.001 |
| IA-stratified help-seeking model | Internet addiction | Informal only vs no help-seeking | 0.843 (0.778-0.913) | <0.001 |
| IA-stratified help-seeking model | Internet addiction | Formal only vs no help-seeking | 1.246 (1.070-1.449) | 0.008 |
| IA-stratified help-seeking model | Internet addiction | Both informal and formal vs no help-seeking | 0.841 (0.691-1.023) | 0.088 |

Notes: IA = internet addiction; PR = prevalence ratio; CI = confidence interval. The outcome was mental health problems, defined as clinically significant depressive symptoms or anxiety symptoms. Poisson regression models with school-clustered CR2 robust standard errors were used. Models adjusted for survey year, sex, age, grade, monitoring site, ethnicity, boarding status, chronic disease history, myopia status, and BMI category. In the main-effect and interaction models, the reference categories were non-addictive internet use and no help-seeking. Interaction terms are shown on the multiplicative PR scale. In the IA-stratified models, no help-seeking was the reference category within each IA status. Formal only refers to adolescents who selected formal source(s) but no informal source; both informal and formal refers to adolescents who selected at least one informal and at least one formal source.

# Table S14. Sensitivity analysis excluding adolescents with formal-only help-seeking for mental health problems.

| Model | IA status | Variable/comparison | Adjusted PR (95% CI) | P value |
| --- | --- | --- | --- | --- |
| Main-effect model | Overall | No Internet use vs non-addictive internet use | 0.626 (0.585-0.669) | <0.001 |
| Main-effect model | Overall | Internet addiction vs non-addictive internet use | 3.631 (3.392-3.887) | <0.001 |
| Main-effect model | Overall | Informal only vs no help-seeking | 0.560 (0.533-0.588) | <0.001 |
| Main-effect model | Overall | Both informal and formal vs no help-seeking | 0.383 (0.346-0.424) | <0.001 |
| Interaction model | Overall | No Internet use vs non-addictive internet use | 0.663 (0.607-0.725) | <0.001 |
| Interaction model | Overall | Internet addiction vs non-addictive internet use | 3.173 (2.944-3.419) | <0.001 |
| Interaction model | Overall | Informal only vs no help-seeking | 0.564 (0.533-0.595) | <0.001 |
| Interaction model | Overall | Both informal and formal vs no help-seeking | 0.382 (0.344-0.425) | <0.001 |
| Interaction model | Overall | No Internet use × Informal only | 0.840 (0.738-0.956) | 0.009 |
| Interaction model | Overall | Internet addiction × Informal only | 1.510 (1.372-1.662) | <0.001 |
| Interaction model | Overall | No Internet use × Both informal and formal | 0.823 (0.614-1.103) | 0.195 |
| Interaction model | Overall | Internet addiction × Both informal and formal | 2.181 (1.732-2.745) | <0.001 |
| IA-stratified help-seeking model | Non-addictive Internet use | Informal only vs no help-seeking | 0.563 (0.533-0.594) | <0.001 |
| IA-stratified help-seeking model | Non-addictive Internet use | Both informal and formal vs no help-seeking | 0.383 (0.344-0.425) | <0.001 |
| IA-stratified help-seeking model | No Internet use | Informal only vs no help-seeking | 0.475 (0.419-0.539) | <0.001 |
| IA-stratified help-seeking model | No Internet use | Both informal and formal vs no help-seeking | 0.315 (0.238-0.418) | <0.001 |
| IA-stratified help-seeking model | Internet addiction | Informal only vs no help-seeking | 0.842 (0.777-0.913) | <0.001 |
| IA-stratified help-seeking model | Internet addiction | Both informal and formal vs no help-seeking | 0.841 (0.691-1.022) | 0.087 |

Notes: IA = internet addiction; PR = prevalence ratio; CI = confidence interval. The outcome was mental health problems, defined as clinically significant depressive symptoms or anxiety symptoms. This sensitivity analysis excluded adolescents with formal-only help-seeking. Poisson regression models with school-clustered CR2 robust standard errors were used. Models adjusted for survey year, sex, age, grade, monitoring site, ethnicity, boarding status, chronic disease history, myopia status, and BMI category. In the main-effect and interaction models, the reference categories were non-addictive internet use and no help-seeking. Interaction terms are shown on the multiplicative PR scale. In the IA-stratified models, no help-seeking was the reference category within each IA status.
