# Supplementary material for: Internet Addiction, Mental Health Help-Seeking, and Mental Health Problems Among Chinese Adolescents: Repeated Cross-Sectional Study
Source: J Med Internet Res. 2026 Aug 12;28:e90014. doi: 10.2196/90014 (PMC13466870; doi:10.2196/90014)
Supplement: Supplementary file 2 [file jmir-v28-e90014-s002.docx]

**STROBE Statement**

STROBE Statement—Checklist of items that should be included in reports of ***cross-sectional studies***

|  | Item No | Recommendation | Yes / No |
| --- | --- | --- | --- |
| **Title and abstract** | 1 | (*a*) Indicate the study’s design with a commonly used term in the title or the abstract | Yes |
|  |  | (*b*) Provide in the abstract an informative and balanced summary of what was done and what was found | Yes |
| Introduction | | | |
| Background/rationale | 2 | Explain the scientific background and rationale for the investigation being reported | Yes |
| Objectives | 3 | State specific objectives, including any prespecified hypotheses | Yes |
| Methods | | | |
| Study design | 4 | Present key elements of study design early in the paper | Yes |
| Setting | 5 | Describe the setting, locations, and relevant dates, including periods of recruitment, exposure, follow-up, and data collection | Yes |
| Participants | 6 | (*a*) Give the eligibility criteria, and the sources and methods of selection of participants | Yes |
| Variables | 7 | Clearly define all outcomes, exposures, predictors, potential confounders, and effect modifiers. Give diagnostic criteria, if applicable | Yes |
| Data sources/ measurement | 8* | For each variable of interest, give sources of data and details of methods of assessment (measurement). Describe comparability of assessment methods if there is more than one group | Yes |
| Bias | 9 | Describe any efforts to address potential sources of bias | Yes. Potential sources of bias were considered and discussed, including social desirability bias in self-reported IA and help-seeking, selection or complete-case bias due to excluded participants with missing data, information bias from screening-based definitions rather than clinical diagnoses, and residual confounding from unmeasured family, peer, school, academic, and treatment-history factors. Participant flow, complete-case analysis, covariate adjustment, and sensitivity analyses were reported to improve transparency. |
| Study size | 10 | Explain how the study size was arrived at | Yes. The study size was determined by the existing provincial school health surveillance system rather than by an a priori statistical sample size calculation. The final analytic sample was obtained after applying the eligibility and complete-case criteria shown in Figure 1. |
| Quantitative variables | 11 | Explain how quantitative variables were handled in the analyses. If applicable, describe which groupings were chosen and why | Yes |
| Statistical methods | 12 | (*a*) Describe all statistical methods, including those used to control for confounding | Yes |
|  |  | (*b*) Describe any methods used to examine subgroups and interactions | Yes |
|  |  | (*c*) Explain how missing data were addressed | Yes |
|  |  | (*d*) If applicable, describe analytical methods taking account of sampling strategy | Yes |
|  |  | (*e*) Describe any sensitivity analyses | Yes |
| Results | | | |
| Participants | 13* | (a) Report numbers of individuals at each stage of study—eg numbers potentially eligible, examined for eligibility, confirmed eligible, included in the study, completing follow-up, and analysed | Yes |
|  |  | (b) Give reasons for non-participation at each stage | No. Reasons for non-participation among invited students were not available in the surveillance dataset. However, reasons for exclusion from the analytic sample were reported in the participant flow diagram. |
|  |  | (c) Consider use of a flow diagram | Yes |
| Descriptive data | 14* | (a) Give characteristics of study participants (eg demographic, clinical, social) and information on exposures and potential confounders | Yes |
|  |  | (b) Indicate number of participants with missing data for each variable of interest | Yes. Missingness was addressed through a complete-case approach, and exclusions due to missing or incomplete data were shown in the participant flow diagram. |
| Outcome data | 15* | Report numbers of outcome events or summary measures | Yes |
| Main results | 16 | (*a*) Give unadjusted estimates and, if applicable, confounder-adjusted estimates and their precision (eg, 95% confidence interval). Make clear which confounders were adjusted for and why they were included | Yes. Observed cell counts and prevalence were reported as unadjusted descriptive estimates, and confounder-adjusted estimates with 95% confidence intervals were reported as the main association estimates. The adjusted covariates were specified in the Methods and table notes. |
|  |  | (*b*) Report category boundaries when continuous variables were categorized | Yes |
|  |  | (*c*) If relevant, consider translating estimates of relative risk into absolute risk for a meaningful time period | No. Not applicable. This repeated cross-sectional study estimated prevalence ratios rather than risks over a defined follow-up period. To aid absolute interpretation, observed cell counts and prevalence were reported in the main tables and figure. |
| Other analyses | 17 | Report other analyses done—eg analyses of subgroups and interactions, and sensitivity analyses | Yes |
| Discussion | | | |
| Key results | 18 | Summarise key results with reference to study objectives | Yes |
| Limitations | 19 | Discuss limitations of the study, taking into account sources of potential bias or imprecision. Discuss both direction and magnitude of any potential bias | Yes |
| Interpretation | 20 | Give a cautious overall interpretation of results considering objectives, limitations, multiplicity of analyses, results from similar studies, and other relevant evidence | Yes |
| Generalisability | 21 | Discuss the generalisability (external validity) of the study results | Yes. Generalisability was discussed, with emphasis on the large provincially representative sample from Shandong Province and caution regarding extension to adolescents in other provinces or countries. |
| Other information | | | |
| Funding | 22 | Give the source of funding and the role of the funders for the present study and, if applicable, for the original study on which the present article is based | Yes |

*Give information separately for exposed and unexposed groups.
